# Polymelia with unhealed navel in an Iranian indigenous young fowl

**Published:** 2017-03-15

**Authors:** Belal Hassanzadeh, Arefeh Rahemi

**Affiliations:** 1 *Central Laboratory, Faculty of Veterinary Medicine, University of Tabriz, Tabriz, Iran; *; 2 *Navid Institute of Radiology and Sonography, Tabriz, Iran.*

**Keywords:** Anomalies, Congenital, Limb bud, Polymelia, Supernumerary limb

## Abstract

Developing supernumerary limbs is a rare congenital condition that only a few cases have been documented. Depending on the cause and developmental conditions, they may be single, multiple or complicated, and occur as a syndrome or associated with other anomalies. Polymelia is defined as the presence of extra limb(s) which have been reported in human, mouse, chicken, calf and lamb. It seems that the precise mechanism regulating this type of congenital malformations is not yet clearly understood. While hereditary trait of some limb anomalies was proven in human and the responsible genetic impairments were found, this has not been confirmed in the other animals especially the birds. Regarding the different susceptibilities of various vertebrate species to the environmental and genetic factors in embryonic period, the probable cause of an embryonic defect in one species cannot be generalized to the all other species class. The present study reports a case of polymelia in an Iranian indigenous young fowl and discusses its possible causes.

## Introduction

Limb malformations are one of the most frequent congenital anomalies of animals and humans. Depending on the cause and conditions, they may be single, multiple or complicated, and occur as a syndrome or associated with other anomalies. Polymelia is defined as the presence of extra limb(s), and depending on its attachment sites to the body could be classified as cephalomelia (attached to the head), notomelia (attached to the vertebral column), thoracomelia (attached to the thorax) and pygomelia (attached to the pelvis).^1^^,^^2^ Although polymelia cases have been reported in human, mouse, chicken, calf and lamb around the world, true polymelia is rarely reported in avian species.^2^^,^^3^ Up to now there are few case reports of chickens with two extra hind limbs such as a 7-week-old slaughterhouse broiler chicken, an 8-week-old Nigerian Nera black chicken and a 7-month-old layer Fayoumi chicken.^4^^,^^5^ The aim of this report is to present the anatomic facts of a case of polymelia in an Iranian domestic young fowl (mixed breed).

## Case Description

**Fig. 1:**
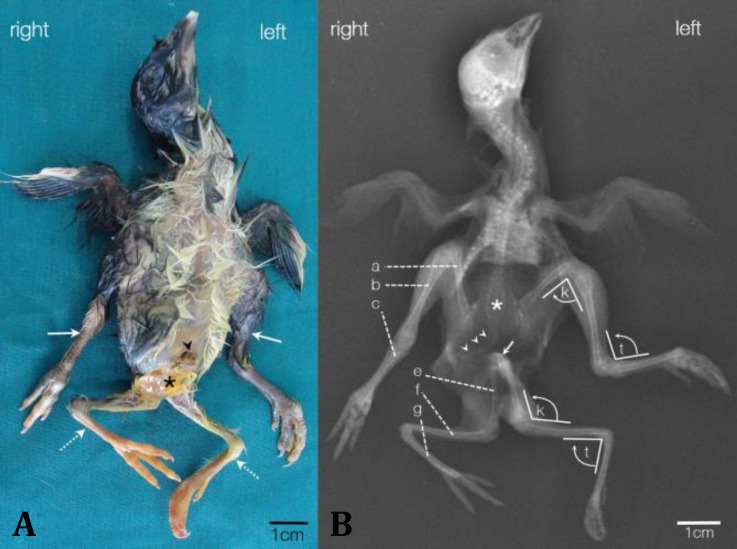
**A. **Ventral view of the polymelia chicken showing the approximately equal size of normal (arrows) and supernumerary legs (dotted line arrows). Compare the color of scales covering the tarsometatarsus and digits. The scales of extra limbs lack typical pigmentation of normal limbs. Note the unhealed navel (arrowhead) and expelled yolk sac contents (asterisk). **B.** X-ray radiograph of the polymelia young fowl (ventrodorsal position) demonstrates the equal number of bones in all normal (upper) and extra (lower) limbs including the femur (a and e), tibiotarsus (b and d), tarsometatarsus (c and f), digits and phalanges. The opposite direction of flexion in the knee (k) and tarsal (t) joints on normal and supernumerary limbs are showed. Note the terminal parts of the spinal column including the synsacrum (asterisk), caudal free vertebrae and pygostyle (arrowheads), and the space between them by the junction of two accessory femurs (arrow). The lower opacity of extra limb bones is distinct in comparison with the normal limb bones

A young fowl with four legs from the rural area of Marand, Iran, was referred to Faculty of Veterinary medicine, Tabriz University. The animal had died before arrival and was kept in the freezer. The gross anatomical characteristics were observed after defrosting and photography was performed from different views. For further studies, the case was transmitted to the Central Laboratory, Faculty of Veterinary Medicine, Tabriz, Iran, at cold temperature (mid-ice cubes). According to the fowl's owner, the chick was about two weeks old and had neither problem in eating and drinking nor lethargy or sickness. The only problem was a difficulty in walking which made it slow in escaping from danger, that maybe cause it trampled down by other animals. Gross anatomical observation revealed two well-developed extra hind limbs (Fig. 1A), consisting of the femur, tibiotarsus, tarso-metatarsus, digits and phalanges, which were attached to the coccygeal area of the body (Fig. 1B). All limbs were intact and no lesion was found on them (Fig. 1A). There were two apparent differences between the normal and accessory limbs. Unlike the normal limbs, the scales of accessory limbs had no pigmentation (Fig. 1A), but the main difference between them was in the knee and tarsal joint. The flexion direction in these joints was the opposite the normal limbs. Postmortem radiological examination confirmed this opposition as well as the lower opacity of supernumerary limb bones (Fig. 1B). Unlike the normal ones the head of extra femurs did not lead to a true hip joint, and they had come together and attached to the coccygeal area. According to the X-ray radiographs, it did not seem that the extra femurs have been attached to any part of the pelvis, pygostyle or free caudal vertebrae (Fig. 1B). Dissociation from the axial skeleton refused the existence of any functional application for these supernumerary limbs and also prevented us from using the term of “pygomelia” for describing the case.

By inspection of the trunk, a yolk sac remnant and an unhealed navel were found in the abdominal cavity but without any sign of inflammation, infection or hemorrhage (Fig. 1A). Thoracic and abdominal viscera were examined and the dark color and vast congestion of lungs were found as the only considerable lesion. This finding besides the signs of a crush on the neck proposed the asphyxia as the main cause of death.

## Discussion

It was best known that all terrestrial vertebrates develop only four limb buds in the embryonic period which are always opposite each other with respect to the midline. Related to the level of Hox gene expression along the anterior-posterior axis, the position of buds is constant in each species. In amphibians, birds and mammals, the fore-limb buds placed at the position of the first thoracic vertebra which is the most anterior expression region of Hoxc-6.

It was also known that some special characteristics of the lateral plate mesoderm induce migration of myoblasts from the somites and entering the limb bud. These characteristics of the lateral plate mesoderm was only found in the limb fields and not elsewhere. Lateral plate mesoderm cells that will become the prospective limb mesenchyme secrete the paracrine fibroblast growth factor 10 (FGF10). This factor has the ability to initiate the limb-forming interactions between the ectoderm and the mesoderm, thus, if beads containing FGF10 are placed ectopically beneath the flank ectoderm, extra limbs emerge. These interactions lead to the formation of the apical ectodermal ridge and its subjacent zone of proliferating activity, both necessary for limb development.^6^ The question is why does the FGF10 ectopic secretion occur in the embryo and what is its regulating mechanisms? Several factors can cause this condition but despite many reports on skeletal system anomalies in domestic animals and birds, it seems that the precise mechanism regulating them is not yet clearly understood.^2^ They could be associated with a wide range of reasons including transgenes, chromosomes, and environmental factors like infectious agents, toxins, techniques involved in fertilization and certain management factors.^7^^-^^9^ In humans, some limb anomalies are inherited and the genes responsible for these anomalies have been identified.^10^^-^^14^ Although there is no strong reason to reject the genetic impairments, recently it has been shown that a higher incubation temperature (about 38.9 ˚C) and higher incubation relative humidity (about 60 to 65%) can increase the rate of malformations including polymelia in domestic fowl.^15^

While the limb buds impairments are considered as the main event leading to the polymelia in birds, some embryonic events could also do this in human. For example, in 2012, a baby boy was born with six legs in Sindh-Pakistan whom extra limbs were diagnosed as remnants of a parasitic twin.^3^ The considerable point of this event was the baby’s father who was an X-ray technician. Anyway, despite the human which a parasitic twin case could end up to polymelia, it cannot be a possible cause of polymelia in birds. Generally, in different vertebrate species the susceptibility to a damaging environmental factor or a certain genetic agent varies in the different stages of bone development.^1^ Therefore, it is a wrong idea to extrapolate a regulating mechanism of such anomalies in one species to the others.

In conclusion, the malformations described in the present paper are a very rare case of polymelia in a chicken involving two hind limbs. Although this is the first published report of avian polymelia in Iran, authors have previously heard of similar cases in some rural areas. Whatever was the cause of this event, chromosomal abnormalities or undesirable incubation conditions, it is extremely valuable to scientific communities to have proper statistics of such anomalies. On the other hand, paying attention to anatomical abnormalities will help to understand their etiology. 
